# Guillain-Barré Syndrome Presenting as Symmetrical Proximal Muscle Weakness: An Atypical Presentation

**DOI:** 10.7759/cureus.50259

**Published:** 2023-12-10

**Authors:** Chris Mehdizadeh, Avrodet Mourkus, Makhlouf Bannoud, Megan Smith, Saad A Ansari

**Affiliations:** 1 Internal Medicine, University of California Riverside School of Medicine, Riverside, USA

**Keywords:** peripheral demyelination, autoimmune, post-infectious etiology, flaccid paralysis, atypical presentation, guillain-barre syndrome (gbs)

## Abstract

Guillain-Barré syndrome occurs via molecular mimicry when a trigger sets off an immune response on peripheral nerve epitopes. Patients typically report an antecedent infection, such as an upper respiratory infection or *Campylobacter jejuni* gastroenteritis. It is typically characterized by progressive, symmetric muscle weakness with absent/decreased deep tendon reflexes. Most cases in the literature report that the paralysis begins in the legs distally and ascends to the extremities. Patients may have sensory symptoms or dysautonomia as well. Notable variant forms include acute motor axonal neuropathy, acute motor/sensory neuropathy, Miller Fisher syndrome, and Bickerstaff brainstem encephalitis. Diagnosis is confirmed if a lumbar puncture shows albuminocytologic dissociation (typically 45 to 200 mg/dL). Nerve conduction studies may also be considered but are not necessary. Management is largely supportive, but intravenous immunoglobulin and/or plasmapheresis for more severe cases may be considered.

## Introduction

Guillain-Barré syndrome (GBS), the most common and severe paralytic neuropathy, has several variants with distinct clinical features. GBS is usually preceded by infection or immune stimulation triggers an autoreactive immune response that targets the peripheral nerves and their spinal roots [1-4]. The most common microorganisms associated with GBS are *Campylobacter jejuni*, Zika virus, and most recently, the SARS-CoV-2 virus [5]. GBS commonly presents as a postinfectious ascending polyneuropathy, with sensory symptoms and weakness that often leads to quadriparesis [6]. The pathologic mechanisms of GBS include axonal damage, demyelination of exons, or combined demyelinating-axonal damage, each form presenting with slightly different pathogenesis and clinical features [7]. The best-known variant is Miller Fisher syndrome, which affects the oculomotor nerves and brainstem by producing antibodies against the GQ1b ganglioside that is involved in these cranial nerve neuromuscular junctions. The treatment for GBS is most often supportive care [7]. Although most patients respond well to immunotherapy, such as intravenous immunoglobulin (IVIG) or plasmapheresis, severe disability due to peripheral nerve damage can occur in 20% of patients, and consequently, even death [4,5]. Diagnosis and management of GBS can also be difficult due to heterogeneity of the disease course and pathogenesis, which hinders early identification and control of infectious triggers, and also lack of GBS progression modifying therapies that limit the rate of nerve damage [8,9].

## Case presentation

**Figure 1:**
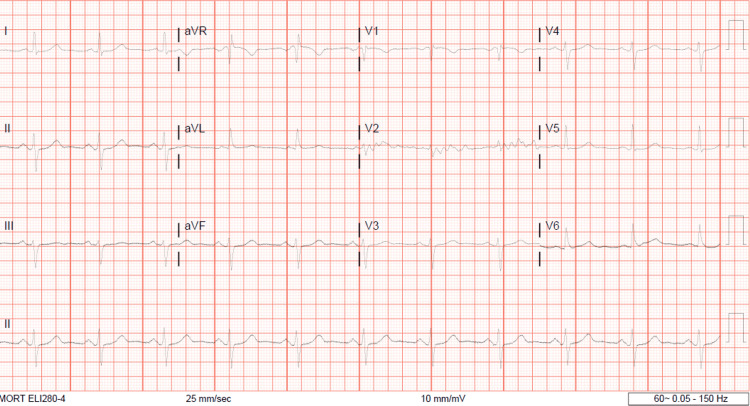
EKG demonstrating sinus rhythm.

We present a 56-year-old man with a history of atrial fibrillation, hypertension, and alcohol use who came in for bilateral proximal upper and lower extremity weakness, tingling, and decreased sensation for three days. He states that he recently had a productive cough approximately 10 days prior to presentation and one day of severe diarrhea three weeks ago. He also mentioned that he has been very stressed lately causing him to drink four to six beers per day. He states that his upper extremity symptoms are more pronounced than his lower extremity symptoms. Physical exam was notable for 3/5 bilateral shoulder and biceps weakness and 4/5 wrist and finger weakness bilaterally. Hip and knee motor exam were 3/5 bilaterally but ankle and toes were 4/5 bilaterally as well. Deep tendon reflexes were diminished diffusely, with the biceps reflex being unobtainable bilaterally. The cranial nerve exam was intact with no deficits and normal speech. He had decreased light touch sensation to the palms, feet, upper torso/chest, and back with paresthesia on his triceps and thighs. The patient was able to walk but was weaker than usual. CT of the head/neck/abdomen/pelvis without contrast were all unremarkable in the ED. Electrolytes, vitamin B9, B12, thyroid panel, creatine kinase, urine drug screening, and vital signs were all within normal limits. C-reactive protein was mildly elevated but the sedimentation rate was normal. He denied any history of diabetes but was found to have a glycosylated hemoglobin (HbA1c) of 13.2%. EKG showed sinus rhythm (Figure 1) and an echocardiogram demonstrated an ejection fraction of 40-50%. The hospitalist team requested an MRI of the head, but the patient denied it as he was claustrophobic. A lumbar puncture was then performed, which showed significant albuminocytologic dissociation (54 mg/dL protein and no elevated nucleated cell count), consistent with GBS using the lumbar puncture albuminocytologic dissociation criteria. Cerebrospinal fluid analysis otherwise had normal color, glucose level, no oligoclonal bands, and normal homocysteine levels. The patient was then started on IVIG infusion (0.4 g/kg) for five days and his symptoms gradually improved back to baseline. By day five, he had a total resolution of his motor symptoms and weakness. He also had resolution of the paresthesia to the triceps and thigh along with resolution of sensory loss to the upper torso/chest and back. He did not, however, regain sensation to light touch of the palms and feet, but given his high HbA1c, this was not investigated further.

## Discussion

GBS is a rare but significant cause of acute muscular paralysis, with the most common variant being acute inflammatory demyelinating polyneuropathy (AIDP). Typically, AIDP manifests as an ascending weakness, often associated with albuminocytologic dissociation in cerebrospinal fluid. While the Miller Fisher syndrome variant has been reported with descending paralysis, these presentations often involve bulbar muscles such as the lateral rectus or other extraocular muscles [2,5]. This case presents a 56-year-old male with an atypical presentation of GBS, emphasizing the heterogeneity of GBS and the importance of considering GBS in patients with acute, worsening muscle weakness, regardless of the pattern of presentation.

The patient's antecedent history of a productive cough and severe diarrhea, along with the presence of significant albuminocytologic dissociation on lumbar puncture, supports the diagnosis of GBS. A prior history of infection makes GBS more likely, but it does not exclude it if there are no identifiable past infections. However, the challenges in diagnosing GBS are due to the atypical presentations of flaccid paralysis, especially when faced with comorbidities that may be potential contributors such as atrial fibrillation, hypertension, and poorly controlled diabetes. The patient's elevated HbA1c of 13.2% indicates poorly controlled diabetes, which may have contributed to the atypical presentation via a polyneuropathic mechanism. It also explains the remaining sensory loss to light touch on the palms and feet, more in line with diabetic neuropathy. His atrial fibrillation and hypertension are known risk factors for cerebrovascular accidents as well.

This patient's history of recent respiratory and gastrointestinal symptoms, as well as the significant albuminocytologic dissociation in the lumbar puncture, corresponds to the reported features of GBS in the literature [10-12]. Typically, both IVIG and plasma exchange were treatment options, with no statistical difference in outcome when compared to each other [2]. Our patient elected to pursue the IVIG treatment option given its ease of administration as compared to plasmapheresis. The response to IVIG infusion in the patient's case aligns with the literature, which underscores the efficacy of this treatment modality in improving symptoms and functional outcomes in atypical GBS presentations [10-12].

A similar atypical case of GBS was reported by Ala Mustafa [10], where a 55-year-old man presented with an ascending upper extremity and descending lower extremity paralysis. This atypical presentation posed a diagnostic challenge and required extensive workup involving imaging studies and ruling out potential differentials. Similarly, a case reported by Shruthi N. Michael [12] described COVID-19-associated GBS with descending paralysis in a four-year-old girl, highlighting the diverse and atypical neurological presentations of GBS. In summary, our case of a 56-year-old man with atypical symmetrical proximal paralysis in GBS exhibits notable distinctions with the literature on atypical presentations as the muscle involvements are not in a lower extremity ascending pattern or bulbar pattern with ophthalmoplegia. Thereby contributing valuable insights to the understanding and management of this rare variant of GBS.

## Conclusions

Our case is unique in that this patient’s presentation did not follow the classic neuropathic pattern of GBS. Rather than having an ascending paralysis, our patient developed a proximal, symmetrical pattern that progressed distally and improved with a five-day course of IVIG. We highlight this case for future providers to consider GBS in any patient with acute, worsening muscle weakness regardless of presentation pattern. Given the availability of lumbar punctures, and their ability to aid in other diagnoses, this procedure should not be delayed once GBS is suspected.
